# *CHD8*-related disorders redefined: an expanding spectrum of dystonic phenotypes

**DOI:** 10.1007/s00415-024-12271-x

**Published:** 2024-03-05

**Authors:** Ugo Sorrentino, Sylvia Boesch, Diane Doummar, Claudia Ravelli, Tereza Serranova, Elisabetta Indelicato, Juliane Winkelmann, Lydie Burglen, Robert Jech, Michael Zech

**Affiliations:** 1 Institute of Human Genetics, School of Medicine, Technical University of Munich, Munich, Germany; 2 Institute of Neurogenomics, Helmholtz Munich, Neuherberg, Germany; 3 Clinical Genetics Unit, Department of Women’s and Children’s Health, University of Padova, Padua, Italy; 4 Center for Rare Movement Disorders Innsbruck, Department of Neurology, Medical University of Innsbruck, Innsbruck, Austria; 5 Sorbonne Université, Service de Neuropédiatrie-Pathologie du Développement, Centre de Référence Neurogénétique, Hôpital Trousseau AP-HP.SU, HU I2D2 Paris, France; 6 Department of Neurology and Centre of Clinical Neuroscience, General University Hospital and First Faculty of Medicine, Charles University, Kateřinská 30, 12 800, Prague, Czech Republic; 7 DZPG, Deutsches Zentrum Für Psychische Gesundheit, Munich, Germany; 8 Munich Cluster for Systems Neurology (SyNergy), Munich, Germany; 9 Cerebellar Malformations and Congenital Diseases Reference Center and Neurogenetics Lab, Department of Genetics, Armand Trousseau Hospital, AP-HP. Sorbonne Université, Paris, France; 10 Developmental Brain Disorders Laboratory, Imagine Institute, INSERM UMR 1163, Paris, France; 11 Institute for Advanced Study, Technical University of Munich, Garching, Germany

**Keywords:** *CHD8*, *CHD8*-NDD, Movement disorders, Dystonia, Exome sequencing, Autism

## Abstract

**Background:**

Heterozygous loss-of-function variants in *CHD8* have been associated with a syndromic neurodevelopmental-disease spectrum, collectively referred to as *CHD8*-related neurodevelopmental disorders. Several different clinical manifestations, affecting neurodevelopmental and systemic domains, have been described, presenting with highly variable expressivity. Some expressions are well established and comprise autism spectrum disorders, psychomotor delay with cognitive impairment, postnatal overgrowth with macrocephaly, structural brain abnormalities, gastrointestinal disturbances, and behavioral and sleep-pattern problems. However, the complete phenotypic spectrum of *CHD8*-related disorders is still undefined. In 2021, our group described two singular female patients with *CHD8*-related neurodevelopmental disorder and striking dystonic manifestations, prompting the suggestion that dystonia should be considered a possible component of this condition.

**Case series presentation:**

We describe three additional unrelated female individuals, each carrying a different *CHD8* frameshift variant and whose clinical presentations were primarily characterized by young-onset dystonia. Their dystonic manifestations were remarkably heterogeneous and ranged from focal, exercise-dependent, apparently isolated forms to generalized permanent phenotypes accompanied by spasticity and tremor. Neurocognitive impairment and autistic behaviors, typical of *CHD8*-related disorders, were virtually absent or at the mild end of the spectrum.

**Conclusions:**

This work validates our previous observation that dystonia is part of the phenotypic spectrum of *CHD8*-related neurodevelopmental disorders with potential female preponderance, raising new challenges and opportunities in the diagnosis and management of this condition. It also highlights the importance of in-depth neurologic phenotyping of patients carrying variants associated with neurodevelopmental disorders, as the connection between neurodevelopmental and movement disorders is proving closer than previously appreciated.

## Introduction

Human *CHD8* is located on chromosomal region 14q11.2. Its main transcript comprises 38 exons encoding for a 290-kDa Chromodomain-Helicase-DNA-binding protein, which acts as an ATP-dependent chromatin remodeler [1]. Through its dynamic interaction with different transcriptional cofactors, which include beta-catenin, CHD7, CTCF, E2F, and p53, the CHD8 protein is involved in upregulating or downregulating the expression of several genes playing pivotal roles in the central nervous system at various stages of development, as well as genes relevant to cell cycle control, epigenetic modifications, and to the proliferation and differentiation of neural progenitor cells and neuroglia [2–4]. Furthermore, CHD8 dosage loss has been shown to be embryonically lethal in animal models [5]. It is therefore unsurprising that loss-of-function variants in *CHD8* have been associated with a spectrum of syndromic neurodevelopmental disorders (*CHD8*-NDD), displaying a wide range of clinical variability, with a yet unexplained higher penetrance in male subjects, and allelic heterogeneity [6–8]. Heterozygous truncating variants are the most common cause of disease, followed by missense variants and inframe insertions/deletions. Reported variants span across the whole length of the gene, without any clear genotype–phenotype correlation recognized to date. The vast majority arise de novo in the probands; however, several familial cases have been documented as well, proving that a proportion of individuals at the milder end of the clinical spectrum may preserve their fitness [9].

The core clinical manifestations reported in individuals harboring *CHD8* pathogenic variants include autism spectrum disorders (ASD) (76% of recorded patients), psychomotor delay, cognitive impairment (68%), postnatal overgrowth with macrocephaly (52%), musculoskeletal defects (79%), abnormal neuroimaging findings (including ventriculomegaly and cerebellar vermis atrophy) (15%), gastrointestinal problems (53%), and behavioral (88%) and sleep-pattern abnormalities (28%) [8]. Seizures, hypotonia, and dysmorphic facial and skeletal features have also been described. However, the growing implementation of non-targeted high-throughput genomic profiling assays, in the forms of whole-exome and whole-genome sequencing analyses, for the diagnosis of patients not primarily referred for neurodevelopmental abnormalities, has been adding new layers to the complexity of *CHD8*-related phenotypes. In 2021, our group described two probands with pathogenic *CHD8* truncating variants and childhood-onset progressive dystonia, suggesting that the spectrum of *CHD8*-related disorders may include a dystonic component, whose characteristics of progressive intensification and generalization are capable of exerting a major impact on the quality of life of affected individuals [10].

Here, we expand our case series by describing three additional, previously unidentified patients with variable dystonic phenotypes harboring heterozygous *CHD8* (likely) pathogenic variants, broadening the clinical spectrum of *CHD8*-related movement disorders, and highlighting the implications in terms of diagnosis and clinical management.

## Methods

In-depth clinical and radiological phenotyping was conducted to assess neurological and systemic abnormalities previously described in *CHD8*-NDD-affected patients from the literature [8]. Whole-exome and whole-genome sequencing was performed on genomic DNA extracted from peripheral leukocytes using previously validated protocols and analysis pipelines [11, 12]. *CHD8* variants were reported according to RefSeq transcript NM_001170629.2; for the predicted protein change, RefSeq NP_001164100.1 was used. Variant interpretation was conducted according to international ACMG/AMP criteria [13]. All patients involved in this case series, or their legal representatives, provided written informed consent as appropriate and in line with ethical guidelines.

## Results

### Case series presentation

Patient 1, a 53-year-old woman, was diagnosed with cerebral palsy soon after birth, initially interpreted as a consequence of birth trauma due to prolonged labor. She was the fifth child of non-consanguineous parents, and family history was unremarkable. A generalized dystonic movement disorder and tremor of the left upper extremity were present since childhood. Infancy was also characterized by delayed psychomotor development, which then transitioned into moderate cognitive impairment without apparent features of progressivity. As an adult, the patient managed to be employed as a sheltered worker, but according to her caregivers, she never reached complete autonomy in her daily living activities. She showed poor response to stressful situations. During her mid-30 s, her dystonic disorder became more aggravating, with increasing symptoms of cervical dystonia and associated pain. At the age of 43 years, she was referred to neurological re-assessment, revealing pronounced right mobile latero-torticollis in the context of a cerebral-palsy syndrome with four-limb muscular hypertonia and distal dystonic posturing (arms > legs). She received symptomatic treatment with tizanidine and botulinum toxin injections, with positive response. The most recent neurological examination also documented truncal dystonia, significant gait instability, postural arm tremor bilaterally (left > right), hyperreflexia, and positive Babinski sign on the right; there were dysarthria, hypermetric saccades, and mild facial dysmorphia with mandibular prognathism. Brain MRI showed no characteristic pathological changes.

Patient 2 was first referred for neurological evaluation as a 25-year-old woman with action-induced upper limb dystonia manifesting since the age of 22 years. Her childhood was unremarkable with normal developmental milestones, but she expressed mild behavioral deficits. She reported having stiffness of the fingers (right > left) and progressive difficulty in performing fine motor tasks. No history for movement disorders was present in her parents and healthy sibling, the only noteworthy information being that her mother was diagnosed with psychiatric disease (schizophrenia). Examination revealed wrist extension and abnormal movements of the fingers III–V when writing. In addition to the writer`s cramp, twisting movements compatible with dystonic episodes of both forearms and hands appeared when performing certain tasks or maintaining a posture. There were no alleviating maneuvers. The patient´s MRI scan was normal, and her physical examination did not detect syndromic features. She was cognitively intact, although her educational level was low. Over a 3-year follow-up, dystonia remained confined to the upper extremities; a trial of levodopa [2 months] was non-beneficial.

Patient 3, a 7-year-old girl with history of mild neurodevelopmental delay and hypotonia, was diagnosed with exercise-induced lower limb dystonia at the age of 3 years. No analogous or otherwise remarkable clinical features were reported in other family members. Examination showed ideo-motor slowness, clumsiness, and fatigability since early childhood. She also presented drooling, which improved by the age of 6 years. At the age of 7, bilateral upper limb dystonic posturing associated with myoclonic jerks was observed, in the absence of ataxia or spasticity. She also had mild intellectual impairment and anxiety disorder. She presented normal growth with slight macrocephaly (+ 2 standard deviations from the mean by age and sex) and mild facial dysmorphia, including elongated face, downslanting palpebral fissures, and large low-set ears. No autistic, sleep-pattern, or digestive abnormalities were noted, and brain MRI did not reveal structural anomalies or basal-ganglia alterations. Extensive routine diagnostic tests were initially unyielding; further lab work-up with determination of her neurotransmitter profile showed mild dopamine and biopterin deficiency, and she was thus treated with levodopa (with partial response). Her condition was gradually progressive over time.

### Molecular analyses

A *CHD8* heterozygous truncating variant was identified in each proband. The analysis performed in patient 1 detected a single-nucleotide insertion c.3524_3525insC, p.(Leu1175Phefs*3), predicted to induce a shift in the open reading frame and a premature stop codon in exon 18. Patient 2 harbored another frameshift variant, caused by a single-nucleotide duplication, c.3832dup, p.(Asp1278Glyfs*2), in exon 19. Patient 3 carried a c.1172dup, p.(Gln392Thrfs*29) frameshift variant in exon 3. All three predicted mutant transcripts are expected to undergo nonsense-mediated mRNA decay, leading to haploinsufficiency in agreement with the well-established monoallelic loss-of-function mechanism in *CHD8*-related conditions [5]. The variants were previously unreported, being absent from major variation archives (ClinVar, LOVD, HGMD) as well as from gnomAD and > 20,000 internal control samples. Genetic analyses performed in the context of the observed clinical features did not reveal any plausible alternative etiologic explanations for the probands´ phenotypes. Segregation analysis confirmed the de novo occurrence of the p.(Gln392Thrfs*29) variant in patient 3, which was therefore classified as “pathogenic” (Class V ACMG) according to PVS1, PM6, and PM2 criteria. Relatives of patients 1 and 2 were unavailable for segregation testing, and therefore, their respective *CHD8* variants were each classified as “likely pathogenic” (Class IV ACMG; PVS1 and PM2 criteria).

Relevant clinical and molecular data collected from the three novel patients hereby described and the two reported previously by Doummar et al. are recapitulated in Table 1.

**Table 1:** Comparison of the phenotypic features of reported *CHD8*-NDD patients in which dystonic manifestation have been described. DBS: deep brain stimulation

	Patient 1	Patient 2	Patient 3	Patient 1 (Doummar et al. 2021)	Patient 2 (Doummar et al. 2021)
Protein variant (heterozygous)	p.(Leu1175Phefs*3)	p.(Asp1278Glyfs*2)	p.(Gln392Thrfs*29)	p.(Arg2217*)	p.(Gly1602Cysfs*5)
Variant origin	Unknown	Unknown	De novo	Unknown	Unknown
Age at onset of the dystonic phenotype	Early childhood	22 yrs	3 yrs	10 yrs	9 yrs
Age at last evaluation	53 yrs	25 yrs	7 yrs	53 yrs	17 yrs
Sex	F	F	F	F	F
Abnormality of prenatal development or birth	Prolonged labor, diagnosis of cerebral palsy	–	Born at term after complicated delivery, no congenital anomalies	–	–
Movement disorders	Generalized dystonia (with progression of cervical dystonia in her mid-30 s), tremor, spasticity	Action-induced limb dystonia, writer´s cramp	Exercise-induced limb dystonia, myoclonic movements, clumsiness, and ideo-motor slowness	Action-dependent involuntary cramps, generalized abnormal muscle contractions, generalized dystonia comprising oromandibular and cervical dystonia, dysdiadochokinesia	Initial neck and upper limb dystonia, progressive generalization to lower limb and axial dystonia
Brain imaging	Normal	Normal	Normal	Flattened caudate nuclei and atrophy of the cerebellar vermis	Slight cerebellar vermis atrophy and bilateral alterations of globus pallidus
Psychomotor development and cognitive function	Delayed psychomotor development, cognitive impairment	Reportedly normal, low educational level	Delayed psychomotor development with hypotonia, mild intellectual disability	Mild intellectual impairment with deficits in verbal fluency and visual working memory	Axial hypotonia, delayed motor milestones; normal intelligence
Autism spectrum disorder or autistic behavior	Not reported/not tested	–	–	–	Speech and nonverbal communication impairment with stereotypic behaviors
Behavioral problems	Social anxiety, inadequate response to stressful situations	Mild	Anxiety disorder	–	Difficulties in social interactions
Dysmorphic signs and growth abnormalities	Prognathism	–	Slight macrocephaly (+ 2 standard deviations) and facial dysmorphia (downslanting palpebral fissures)	–	CC > 98th percentile, high forehead, supraorbital ridge, and pointed chin
Current/most effective medication	Tizanidine for spasticity, botulinum toxin (I.M. injection) for dystonia	Levodopa without effect	Levodopa (partial response)	DBS	DBS

## Discussion and conclusions

Since its identification as a morbid gene in 2012 [6], the role of *CHD8* in neurodevelopmental disorders has been thoroughly investigated, with more than a hundred cases affected by pathogenic variants reported to date. An extreme phenotypic variability, in terms both of range and intensity of expressivity of clinical manifestations, has been highlighted to date. In this landscape, movement abnormalities are beginning to emerge as an under-recognized component. In a recent extensive review of *CHD8*-NDD cases, “involuntary movements” were reported in 17% of patients in whom this aspect could be assessed [8]. However, very few well-characterized instances have so far been described, limiting our understanding of the actual relevance of such manifestations in the overall clinical presentation and management of *CHD8*-NDD patients. The case series presented in this work validates the observation, made by Doummar et al. in 2021, that *CHD8* heterozygous loss-of-function variants can be associated with phenotypes prominently characterized by young-onset dystonia, with mild-to-moderate, in some cases even barely detectable signs of neurocognitive disorders [10]. Despite the limited number of cases, a heterogeneous spectrum of dystonic manifestations has already been recorded, ranging from focal to generalized, from paroxysmal or action-induced to permanent, from apparently isolated to complex with spasticity and cerebral palsy-like pictures. *CHD8*-related dystonia appears to be progressive: focal, paroxysmal, or action-induced dystonic phenotypes were observed mostly in younger probands, often paired with mild neurobehavioral abnormalities and developmental milestones delay; on the other hand, patients with a sufficiently long documented history of disease, namely both individuals from the Doummar series and Patient 1 from this work, showed increasingly aggravating, painful, and poorly controlled manifestations, including tremor and spasticity. Although dystonic tremor and essential tremor are often difficult to discriminate [14], tremor in *CHD8* patients was observed only in the context of generalized dystonia/cerebral palsy-like pictures and in body parts that were also affected by dystonia, and it was therefore classified as a dystonic tremor by movement disorder experts.

Although it is possible that dystonia is an overall rare manifestation across the spectrum of *CHD8*-related disorders, the low rate of cases so far reported in the literature could also partly be attributable to referral biases, as most case series involved patients primarily referred for ASD, the severity of which could have hindered proper assessment of movement patterns and possible associated clinical comorbidities. Furthermore, as highlighted by our series, some of the most debilitating motor manifestations arose in late childhood/early adulthood, when patients may have already been lost for follow-up. At the same time, our series suffers from a bias on its own, as all our patients were referred primarily for their dystonic manifestations. Further studies on larger, unbiased case series are necessary to determine the actual prevalence of *CHD8*-related movement disorders. Careful, focused motor system re-assessment of previously published cases may also provide further insight into the topic.

Establishing valid genotype–phenotype correlations in *CHD8*-NDD has so far proved to be a complex effort, due to the apparently even distribution of causative variants across the whole gene and the overall higher prevalence of truncating variants [8, 15]. This observation seems consistent with our study group, as all identified variants were truncating and were predicted to result in haploinsufficiency, leading to comparable effects on protein expression. Hence, it could be hypothesized that factors other than allelic variations in *CHD8*, whether genetic or non-genetic, could have more significant relevance in this context.

An interesting observation in this regard can be made about the gender of our *CHD8*-dystonic probands. As opposed to the overall prevalence of *CHD8*-related ASD, which is characterized by a solid 2:1 male-to-female ratio despite the autosomal dominant mode of transmission of the causative gene [8], all dystonic *CHD8* patients from our case series and the one from Doummar et al. happened to be females. With all due caution relative to the small sample size and the uncertainty ascribable to the possible incomplete phenotyping of male *CHD8*-NDD patients reported so far in the literature, the available data suggest a possible gender unbalance with regards to *CHD8*-related dystonia. The hypothesis that dystonia is more common in *CHD8* female patients than in their male counterparts would be further corroborated by the anecdotal observation that neither of the two male sons of the adult dystonic patient described by Doummar et al. showed any sign of movement disorder, despite carrying the same causative variant of the mother and exhibiting an otherwise penetrant phenotype, with manifest signs of neurodevelopmental impairment. If confirmed, such an oppositely skewed distribution of ASD and dystonia between males and females in *CHD8* patients could represent an interesting model for both the long speculated female protection effect in ASD [16], and the increased prevalence of specific forms of idiopathic dystonia commonly observed in female patients from the general population [17].

The confirmation of movement disorders as a part of the *CHD8* phenotypic spectrum, to a point where dystonia can even be the most prominent and earliest manifestation of *CHD8*-related disorder, prompts a series of further considerations. First it strongly raises the question of whether a neurologic evaluation focused on the assessment of possible movement disorders should be included in the first-line clinical management of all newly diagnosed *CHD8* patients and be proposed at least once to the ones already in follow-up. Second, it remarks the potential diagnostic weakness of targeted custom gene panels in the investigation of neurologic disorders, as even apparently isolated forms could mask more complex, syndromic conditions, leading to possible delays in providing the correct diagnosis for the disease and the subsequent counseling to the proband and their family. Finally, it highlights the importance of continuing pursuing a more and more comprehensive neurologic phenotyping of patients harboring genomic variations which have so far been primarily associated with neurodevelopmental disorders. This consideration is especially relevant regarding genes involved in regulatory processes or showing a higher expression in the central nervous system even in postnatal life [18]. The pathophysiological bases of motor system involvement in *CHD8*-related disorders are still unclear; however, the characterization of important CHD8 targets relevant to movement disorders such as beta-catenin and *SCN2A* [19], the recurrent identification of cerebellar structural abnormalities in probands [8, 10], and recent animal experiments highlighting a crucial role for CHD8 in extrapyramidal nervous structures’ development [20], represent promising research prospects for investigating the etiological plausibility of this connection.

In conclusion, we characterized three additional unrelated cases of syndromic and non-syndromic movement disorder patients harboring *CHD8* pathogenic or likely pathogenic variants, confirming our previous observation that movement disorders should be considered a non-neglectable component of the *CHD8*-NDD spectrum and emphasizing the increasingly evident connection between movement and neurodevelopmental disorders.

## Data Availability

The data presented in this study are available on request to the corresponding author.

## Abbreviations

***CHD8-NDD***: CHD8-related neurodevelopmental disorders
**ASD**: Autism spectrum disorders
